# A family of long intergenic non-coding RNA genes in human chromosomal region 22q11.2 carry a DNA translocation breakpoint/AT-rich sequence

**DOI:** 10.1371/journal.pone.0195702

**Published:** 2018-04-18

**Authors:** Nicholas Delihas

**Affiliations:** Department of Molecular Genetics and Microbiology, School of Medicine Stony Brook University, Stony Brook, New York, United States of America; INSERM UMR S_910, FRANCE

## Abstract

FAM230C, a long intergenic non-coding RNA (lincRNA) gene in human chromosome 13 (chr13) is a member of lincRNA genes termed family with sequence similarity 230. An analysis using bioinformatics search tools and alignment programs was undertaken to determine properties of FAM230C and its related genes. Results reveal that the DNA translocation element, the Translocation Breakpoint Type A (TBTA) sequence, which consists of satellite DNA, Alu elements, and AT-rich sequences is embedded in the FAM230C gene. Eight lincRNA genes related to FAM230C also carry the TBTA sequences. These genes were formed from a large segment of the 3’ half of the FAM230C sequence duplicated in chr22, and are specifically in regions of low copy repeats (LCR22)s, in or close to the 22q.11.2 region. 22q11.2 is a chromosomal segment that undergoes a high rate of DNA translocation and is prone to genetic deletions. FAM230C-related genes present in other chromosomes do not carry the TBTA motif and were formed from the 5’ half region of the FAM230C sequence. These findings identify a high specificity in lincRNA gene formation by gene sequence duplication in different chromosomes.

## Introduction

Long non-coding RNA (lncRNA) genes make up a major portion of the human genome [1] and tens of thousands of lncRNA transcripts have been detected [2, 3]. There has been a major effort to characterize and understand the origin and historical lineage of this genetic information. Characterizing this large amount of genes and transcripts is daunting, but significant progress has been made (see references [4–7] for a partial list). The origins of lncRNA genes are being addressed as to the de novo formation of lncRNA genes, the formation of lncRNA genes via gene duplications, formation of functional pseudogenes from protein coding genes or derivation from enhancer sequences [8–12].

In this study, we analyzed a family of lncRNA genes related to the long intergenic non-coding RNA (lincRNA) gene FAM230C and address gene composition and origins. Although functions of RNA transcripts from this family are not known, we discovered that at the DNA level, many of the genes carry a prominent DNA translocation breakpoint motif and that these genes are formed and concentrated in a fragile chromosomal region in human chromosome 22 (chr22), 22q11.2.

22q11.2 is a region that displays a high frequency of chromosomal translocation [13], and it can undergo deletions and other chromosomal abnormalities that result in disease consequences [14]. This region contains multiple copies of the repeat element termed Translocation Breakpoint Type A sequence (TBTA), which contains several sections of AT-rich and highly variable sequences. TBTA and its related sequences play a role in translocation via the palindromic AT-rich repeat sequence (PATRR) [13, 15–18]. In addition a flanking AT-rich region of the Translocation Breakpoint sequence is also associated with translocation activity [18].

Here we show that the lincRNA gene FAM230C, which is on chromosome 13 (chr13), carries the TBTA/AT-rich motif in its sequence. More significantly, eight-related lincRNA genes that stem from copies of the FAM230C sequence also carry the motif. These genes are formed and present only in chr22 and they are exclusively in low copy repeats (LCR22)s situated within or close to the 22q11.2 region [19, 20]. LCR22 segmental duplications are thought to participate in nonallelic homologous recombinations, leading to 22q11.2 deletions [19, 20]. Findings reported here on lincRNA genes harboring AT-rich repeat sequences parallels protein gene intron sequences known to harbor purine/pyrimidine (Pu/Py) repeat elements [21].

A total of seventeen lncRNA genes are found in various chromosomes that originate from different segments of the FAM230C sequence, of which nine do not carry the TBTA. Here we propose that the FAM230C sequence serves as a pool for formation of diverse lncRNA genes by sequence duplication and subsequent modification.

With respect to RNA transcription, data provided by NCBI on RNA-seq transcript expression from the eight FAM230C-related genes on chr22 show that RNA transcripts are expressed almost exclusively in the testes (https://www.ncbi.nlm.nih.gov/gene/) [22]. Thus there is a high specificity with these lincRNA genes in terms of the incorporation of a DNA translocation motif and the formation in a specific chromosomal location, as well as in RNA expression that is in a selective tissue.

## Materials and methods

### Nucleotide sequence sources

Translocation Breakpoint Type A sequence [13] is from GenBank: AB261997.1, NCBI website: https://blast.ncbi.nlm.nih.gov/Blast.cgi.

Alu sequences are from the Dfam website http://Dfam.org. [23, 24].

Human Satellite I sequence is from NCBI GenBank: X00470.1 [25, 26]. Human Satellite1 is part of a group of repeat sequences found in centromeres of chromosomes [27].

Human chr22 sequence is from NCBI, Homo sapiens chromosome 22, GRCh38.p7 Primary Assembly, Sequence ID: NC_000022.11

### Data bases for lncRNA genes

lincRNA gene sequences, exon and intron specifications, and chromosomal coordinates are from the Ensemble Genome Browser, http://useast.ensembl.org/Homo_sapiens/Info/Index [28] the Vega website, http://vega.sanger.ac.uk, version 68 and NCBI, https://www.ncbi.nlm.nih.gov/gene. Additional sites employed for lncRNA gene specifications are: Gene Cards, http://www.genecards.org and HUGO Gene Nomenclature Committee, https://www.genenames.org. As multiple names are still used for lncRNA genes, both the Ensemble/Vega and the NCBI/ names are used in this paper. Ensemble/Vega coordinates for lncRNA genes are listed in this manuscript. The Vega Browser will be retired in 2020 and meshed with the Ensemble Genome Browser.

### Nomenclature used for eight genes that carry the TBTA motif, are only in chr22 and originate from the 3’ end of the FAM230C sequence

1. LINC01663
  Ensemble: LINC01663 ENSG00000276095
  NCBI: LINC01663 NCBI ID: 100996432
  Vega: AC008103.3 OTTHUMG00000188102
2. LINC01660 (AC011718.2)
  Ensemble: LINC01660 ENSG00000274044
  NCBI: LINC01660 NCBI ID: 729461
  Vega: AC011718.2 OTTHUMG00000188347
3. LINC01662 (AC008132.15)
  Ensemble: LINC01662 ENSG00000182824
  NCBI: LINC01662 NCBI ID: 642643
  Vega: AC008103.3 OTTHUMG00000187471
4. FAM230B
  Ensemble: FAM230B ENSG00000215498
  NCBI: FAM230B NCBI ID: 642633
  Vega: FAM230B OTTHUMG00000150782
5. AP000552.1 (KB-1183D5.13)
  Ensemble: AP000552.1 ENSG00000206142
  NCBI: LOC100996335 NCBI ID: 100996335,
  Vega: KB-1183D5.13 OTTHUMG00000150795
6. AC007731.1
  Ensemble: AC007731.1 ENSG00000188280
  NCBI: LOC101927859 NCBI ID: 101927859,
  Vega: AC007731.1 OTTHUMG00000150686
7. AC008079.1
  Ensemble: AC008079.1 ENSG00000187979
  NCBI: LOC100996415 NCBI ID: 100996415
  Vega: Not characterized
8. LINC01658 (AP000345.1)
  Ensemble: LINC01658 ENSG00000178248
  NCBI: LINC01658 NCBI ID: 388882,
  Vega: AP000345.1 OTTHUMG00000150669

### ncRNA genes that contain FAM230C sequences but do not carry the TBTA

1. AP000552.3 ENSG00000237407; KB-1183D5.14 OTTHUMG00000150793 (chr22)
2. FAM230A NCBI ID 653203; UCSC:ID uc062bir.1 (chr22)
3. AP003900.1 ENSG00000277693 (chr21)
4. EIF3FP1 NCBI ID: 54053 (chr21)
5. EIF3FP2, NCBI ID:838880 (chr13)
6. EIF3FP3 NCBI ID: 339799, (chr2)
7. DUXAP9 ENSG00000225210 (chr14)
8. DUXAP10 ENSG00000244306 (chr14)
9. CECR7 ENSG00000237438 (chr22)

Genes # 3–9 carry sequences from the 5’ half of FAM230C, genes # 1 and 2 from the 3’ half of FAM230C. As more lncRNA genes are annotated and characterized, this number may increase.

### Sequence alignment methods and reverse complement determinations

For alignment of two or more nucleotide sequences, the EMBL-EBI Clustal Omega Multiple Sequence Alignment program, website: http://www.ebi.ac.uk/Tools/msa/clustalo/ was used. For alignment of two sequences showing alignment with a reverse complement sequence, the NCBI Basic Local Alignment Search Tool for two or more sequences was used with default parameters. The Percent identity between two sequences was determined by the NCBI Basic Local Alignment Search Tool. To determine the reverse complement of nucleotide sequences, The Sequence Manipulation Suite was used, website: http://www.bioinformatics.org/sms/rev_comp.html

### Satellite/Alu/AT-repeat identification

RepeatMasker analysis [23, 24] was used to determine the presence of satellite/Alu/AT-rich repeats in genomic sequences.

The Dfam RepeatMasker website is: http://www.repeatmasker.org/cgi-bin/WEBRepeatMasker. Search Engines used were abblast and rmblast. The display of repeat signatures for gene sequences, as determined by RepeatMasker is a useful addition to sequence alignments, as the presence of repeat sequences and low complexity sequences provides ambiguity.

### Genomic searches

To find sequences similar to Translocation breakpoint Type A and FAM230C, the blast search engine was used with the following website:

NCBI Blast, website: https://blast.ncbi.nlm.nih.gov/Blast.cgi?CMD=Web&PAGE_TYPE=BlastHome

The databases targeted were Human genomic + transcript, reference genomic sequences. The parameters were default settings as well as parameter Optimize for Highly similar sequences.

To find lncRNA genes that have sequences similar to a lncRNA gene sequence, the Ensemble BLAT/BLAST Search Engine was used: (http://useast.ensembl.org) [28]. BLAT was the search tool. Parameters used: The General options, Scoring options, Filters and masking options were the default parameters, except for BLASTN where no filtering for low complexity sequences was used.

### RNA transcript analyses

The data in Supporting Information, S1 Fig and S1 Table showing RNA transcript expression levels from the eight FAM230C-related lincRNA genes were obtained from the NCBI RNA transcript analysis and are posted on webpage: https://www.ncbi.nlm.nih.gov/gene/ [22] under the project title: HPA RNA-seq normal tissues. Data can be accessed by including the gene name in the search query. S1 Table, which shows tissue locations and RPKM values of RNA transcripts is a compilation of data from the NCBI website.

## Results

### Translocation Breakpoint Type A sequence Analysis

**Fig 1:**
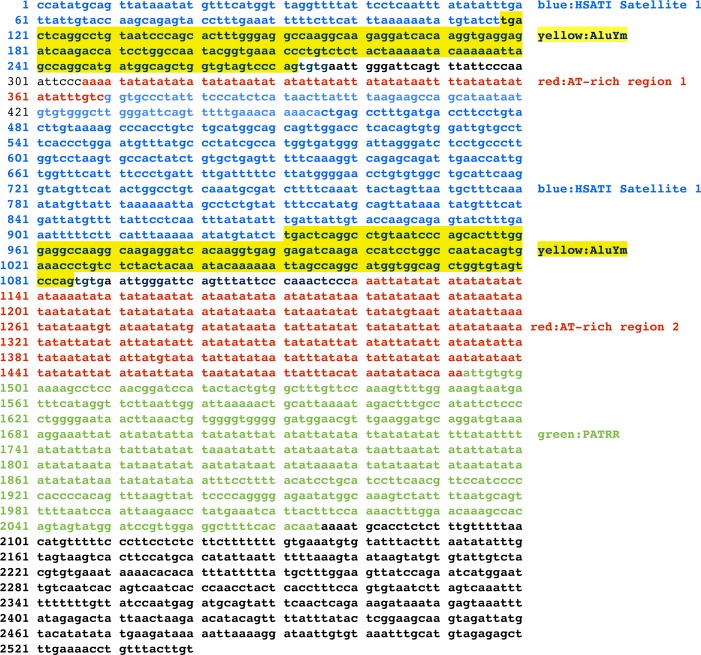
The sequence of the Translocation Breakpoint Type A (TBTA) is from GenBank sequence ID:AB261997.1 [13]. The figure shows the HSATI, Alu, AT-rich regions and the PATRR. Positions 1–306 represent a direct repeat of 814–1119. Positions of Satellite/Alu/AT-rich regions in the translocation breakpoint type A sequence were determined by RepeatMasker analysis).

Central to variability and expansion of AT-rich sequences in chromosome 22 is the repeat element Translocation Breakpoint Type A, NCBI GenBank accession: AB261997.1 [13, 16]. This element carries a complex combination of diverse motifs: two partial copies of a satellite HSATI sequence, two copies of a fragment of an Alu sequence similar to subspecies AluYm, two redundant AT-rich sequences termed 1 and 2, and a palindromic AT-rich repeat, the palindromic translocation breakpoint hot spot sequence (PATRR) (Fig 1). Table 1 shows a RepeatMasker analysis of the TBTA sequence, with start and end positions of the satellite/alu/AT-rich motif in the TBTA. HSATI and Alu elements have previously been shown to be part of a related translocation breakpoint sequence [15, 29]. In addition, sequences that form exon1 of several lncRNA genes as well as introns of protein genes have a high similarity to the translocation breakpoint sequence and appear to have originated from the TBTA [30]. Thus the TBTA carries multiple elements, but importantly for the translocation process, regions of high AT-variable sequences.

**Table 1:** Satellite/Alu/AT-rich positions in TBTA.

Start	End	Satellite/Alu /AT-rich	Class/family*
1	117	HSAT1	Satellite
118	272	AluYm1	SINE/Alu
310	364	(AT)n	Simple repeat
370	930	HSAT1	Satellite
931	1085	AluYm1	SINE/Alu
1124	1469	(TA)n	Simple repeat
1686	1880	(ATTATAT)n	Simple repeat

*Data from RepeatMasker analysis of sequence from NCBI GenBank: AB261997.1

In terms of origin, the TBTA sequence has an 85% identity with Satellite 1 subspecies, Human Satellite I (NCBI GenBank: X00470.1), which is described as a sequence that “includes a male specific 2.47 kb tandemly repeated unit containing one Alu family member per repeat” [25]. As to signatures, the TBTA also mimics the signatures of Human Satellite I. RepeatMasker analysis of Human Satellite 1 shows a pattern of satellite/Alu/AT-repeat (Table 2). This is the similar pattern found in the TBTA (Table 1), This suggests that the HSAT1/Alu/AT-rich motif in the TBTA originated from a satellite sequence, confirming the original findings of Babcock et al for a related translocation breakpoint sequence [15, 29]. An unidentified satellite sequence related to Human Satellite I might have provided the signatures of the TBTA.

**Table 2:** Satellite/Alu/ Repeat in human satellite I.

Start	End	Satellite/Alu /repeat	Class/family*
1	505	HSAT1	Satellite
506	792	AluSc8	SINE/Alu
796	903	(TATATGT)n	Simple repeat

*Data from RepeatMasker analysis of sequence from NCBI GenBank: X00470.1.

These signatures are useful for the identification of TBTA-related sequences, as there can be an ambiguity in alignment of nucleotide sequences due to the internal repeats and the low complexity of AT-rich regions displayed by the TBTA/AT-rich element. Thus the satellite/Alu/AT-rich signature is used with the lincRNA gene analyses discussed here.

### lincRNA gene FAM230C contains copies of the TBTA/AT-rich motif

FAM230C is a gene termed Homo sapiens family with sequence similarity 230 member C, long intergenic non-coding RNA (NCBI Gene ID is 26080). The nomenclature by Vega is RP11-341D18.3 OTTHUMG00000189381 and by Ensemble as FAM230C ENSG00000279516. This gene consists of 37,928 bp and is present in chromosome 13 with coordinates chr:13:18194697–18232624. It has 8 exons and its transcript is considered a processed transcript but with unknown function.

FAM230C contains 3 copies of the HSAT1/Alu/AT-rich sequence of the TBTA. An analysis by RepeatMasker of the region of the FAM230C gene that contains the TBTA shows the similar signature pattern, the HSAT1/Alu/AT-rich sequence with the HSAT1/Alu/AT-rich motif repeated three times in FAM230C albeit there are minor differences involving two Alu subspecies (Table 3). The three consecutive repeats also include an extra HSAT1 sequence. These TBTA-related sequences are approximately in the middle of the FAM230C sequence, encompass nt positions 17010–22032 of FAM230C gene (the FAM230C gene is 37928 bp) and they reside in intron 1 of the FAM230C lincRNA.

**Table 3:** Satellite/Alu/AT-rich regions of FAM230C, positions 17010–22032*.

Start	End	Satellite/Alu /AT-rich #	Class/family	Repeat
17010	17576	HSATI	Satellite	1
17577	17854	AluSc8	SINE/Alu	
17864	18258	(TA)n	Simple repeat	
18259	18824	HSATI	Satellite	2
18825	19117	AluSc8	SINE/Alu	
19118	20117	(TATATTA)n	Simple repeat	
20119	20607	HSATI	Satellite	3
20610	20761	AluYm1	SINE/Alu	
20800	20952	(TA)n	Simple repeat	
21862	22032	HSATI	Satellite	

*Data from RepeatMasker analysis of sequence of FAM230C

**Fig 2:**
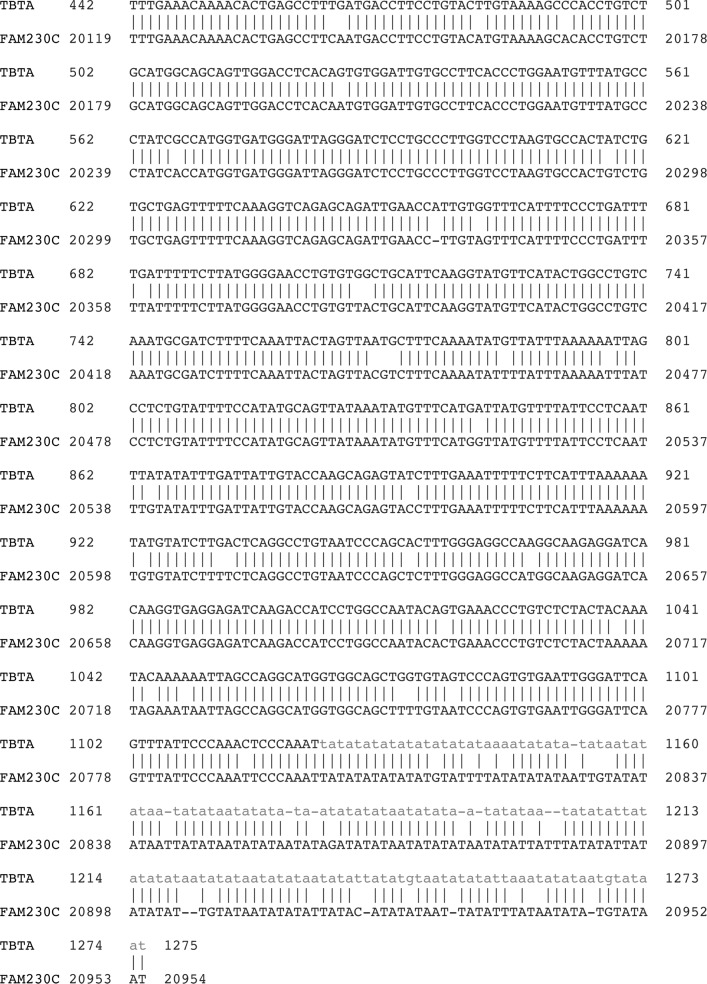
Alignment of the TBTA sequence with lincRNA gene FAM230C sequence, which encompasses repeat #3 in FAM230C (Table 3). The nucleotide positions in both sequences are shown. The NCBI Align Two Sequences Nucleotide BLAST program was used.

An alignment of the TBTA sequence with the sequence of repeat #3 in FAM230C is in Fig 2. The similarity in sequence that is shown in Fig 2 extends from the HSAT1 (position 442 of the TBTA), encompasses the Alu sequence, and includes part of the AT-rich region #2 up to position 1275 (see Fig 1). The identity between the two sequences is 93%. The alignment also shows the variability in sequence in the AT-rich region. Thus the signature pattern and a segment of the TBTA nucleotide sequence are both present in the FAM230C gene.

### Sequences of lincRNA gene FAM230C, including the TBTA are present in eight-related lincRNA genes in chr22

**Fig 3:**
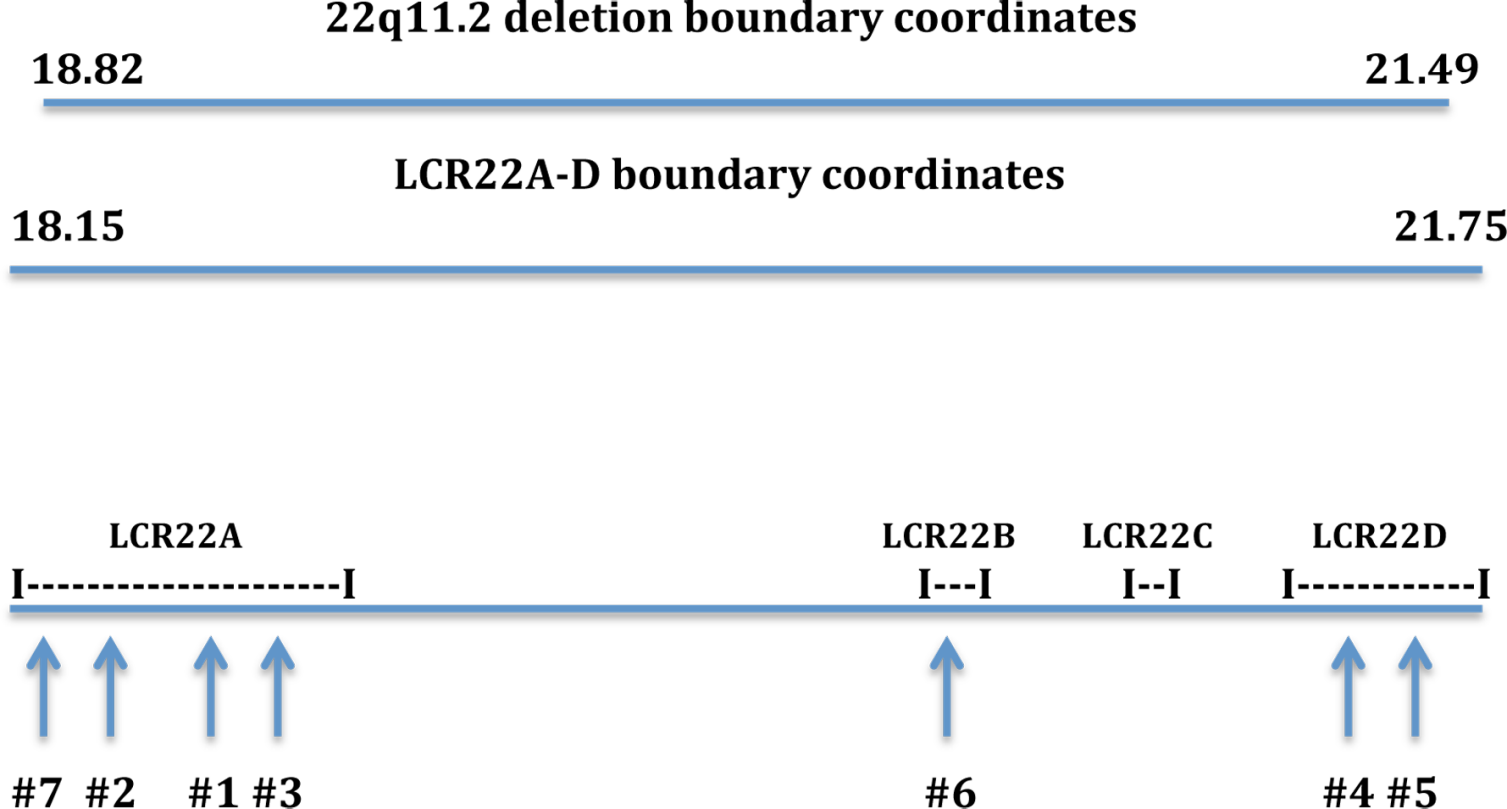
Diagrammatic representation of the 22q11.2 region showing positions of LCR22A-D and lincRNA genes #1–7 that carry the TBTA and are listed in Materials and Methods. 22q11.2 coordinates are from Guna et al [31] and LCR22A-D coordinates from Demaerel et al [32]. Line drawings that represent chromosomal distances, LCR22 positions and lincRNA gene positions are approximate.

Blast/Blat searches using the Ensembl genome browser (http://useast.ensembl.org) were employed to look for genes that display an identity with FAM230C; the FAM230C sequence was used as the query. Two groups of genes showed positive results. One group has an identity with the 3’ half of the sequence, starting at position ~17000 bp that includes the TBTA/AT-rich repeat of FAM230C, and another that has identity with the 5’ end of FAM230C but does not contain TBTA sequences. All FAM230C-related lincRNA genes that display the TBTA sequence and its satellite/Alu/AT-rich signature are in chr 22, in or near the 22q11.2 deletion region (coordinates chr22:18,820,303–21,489,474) [31]. Seven genes are within one of the low copy repeats (LCR22A-D) in 22q11.2 (LCR22A-D span coordinates chr22:18,150,000–21,750,000) [32] (Fig 3). The eighth gene, AP000345.1 (LINC01658) is in LCR22F (formally, LCR22-6’, chr22:23306926–23679116.) [20] (personal communication, Deyou Zheng). LCR22F (LCR22-6’) is close to but outside of the 22q11.2 region.

Table 4 shows the eight genes that have a high similarity with the 3’ segment of FAM230C and harbor the TBTA-AT-rich sequences. Gene lengths, nt positions analogous to FAM230C positions and gene locations in low copy repeats (LCR22A-D and LCR22F) [19, 20, 29, 32] in 22q11.2 are shown. Chromosomal coordinates for these genes are from Homo sapiens chromosome 22, GRCh38.p7 Primary Assembly, Sequence ID: NC_000022.11. All eight FAM230C-related lincRNA genes show an identity with the 3’ half sequence of FAM230C from nt positions ~17000–37928, which includes the TBTA sequences (FAM230C positions nt 17010–22,032).

**Table 4:** Properties of lincRNA genes in chr22.

lincRNA gene, chromosomal location,	lincRNA nt positions	FAM230C nt positions
length; LCR22 position		
1. LINC01663 (AC008103.3)	314 -21974	17003- 37928
chr22: 18,872,943-18,895,007		
22065 bp; LCR22A		
2. LINC01660 (AC011718.2)	4028-21304	16903-37928
chr22: 18,361,223-18,391,705		
30,483 bp; LCR22A		
3. LINC01662 (AC008132.15)	1-16346	17872-37928
Chr22: 18,733,314-18,758,506		
25,913 bp LCR22A		
4. FAM230B	1-16529	17410-37928
chr:22: 21,167,158-21,192,756		
25,599 bp; LCR22D		
5. AP000552.1 (KB-1183D5.13)	1-16588	17408- 37928
chr22: 21,300,390-21,325,642		
25,253 bp; LCR22D		
6. AC007731.1	1- 16551	17401-37928
chr22:20,338,205-20,354,972		
16,768 bp; LCR22B		
7. AC008079.1	380- 16994	16994-37928
chr: 22: 18,177,438-18,206,515		
29,078 bp; LCR22A		
8. LINC01658 (AP000345.1)	1- 16761	16668-37928
chr22: 23461486-23487580		
26,095 bp; LCR22F		

**Fig 4:**
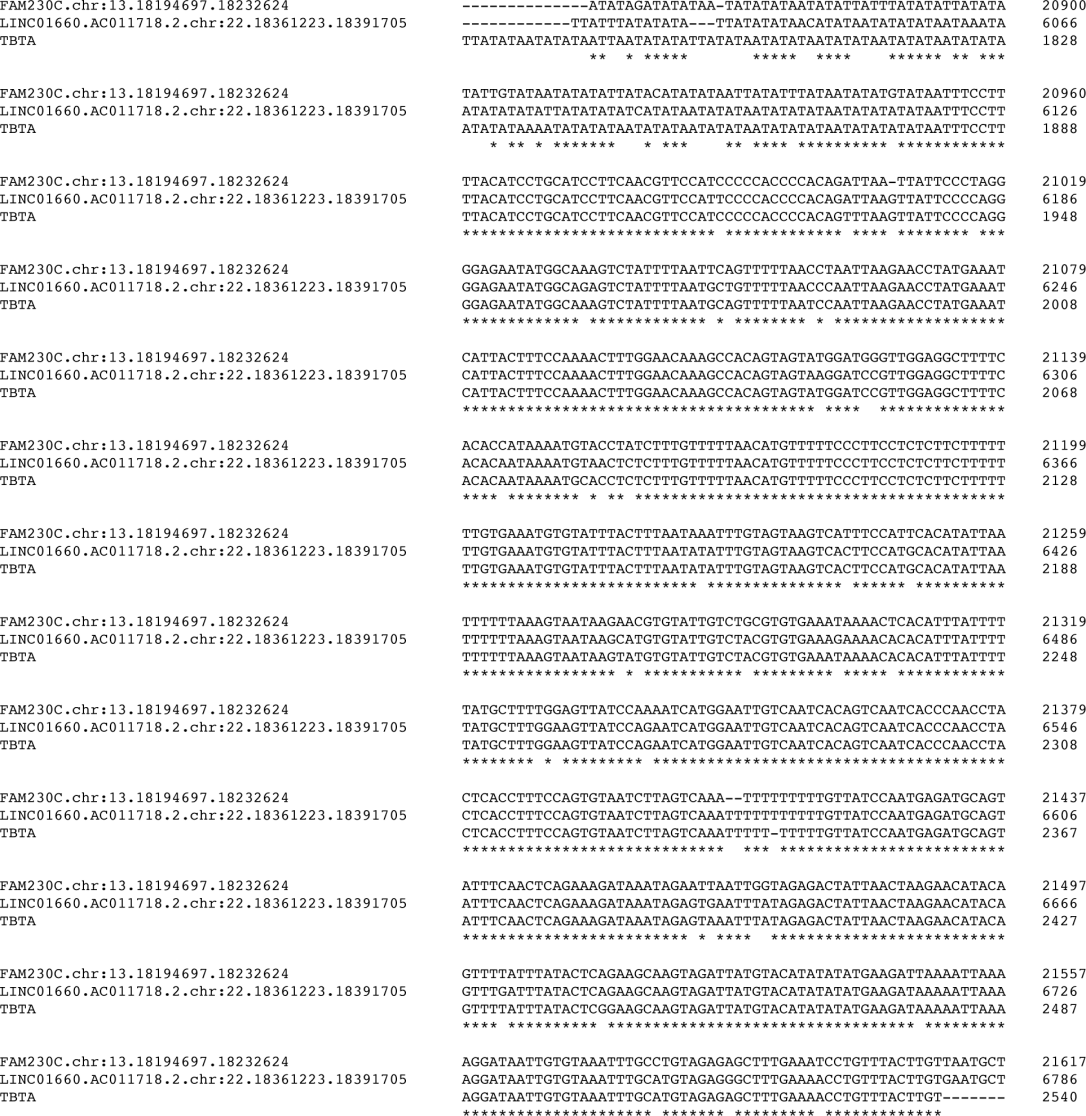
Sequence alignment of LINC01660 (AC011718.2) lincRNA gene sequence with FAM230C and TBTA sequences. sequences. The figure shows the similarity of the LINC01660 (AC011718.2) sequence with that of the TBTA and extends from approximately the middle of the PATRR (TBTA position 1686) to the 3’ end of the TBTA (position 2540). Excluding the AT-variable sequences and a PATRR sequence rearrangement, the entire TBTA sequence is found in LINC01660. Clustal Omega Multiple Sequence Alignment program (EMBL-EBI) was used for alignment.

As an example, presence of the HSAT1/Alu/AT-rich repeat signature in lincRNA gene LINC01660 (AC011718.2) is shown in Table 5. There are two complete repeats of the HSAT1/Alu/AT-rich motif present. The start of the repeat motif is close to the 5’ end of this gene and begins in intron 3, but unlike FAM230C, the HSAT1/Alu/AT-rich repeat also encompasses two exons. The other lincRNA genes listed in Table 4 also have the HSAT1/Alu/AT-rich repeat that encompass an exon. The alignment of FAM230C and TBTA nucleotide sequences with the sequence of LINC01660 (AC011718.2) is in Fig 4. It shows the high similarity of the LINC01660 sequence with the Satellite/Alu/AT-rich repeat #3 sequence of FAM230C. Nt positions 6020–6103 of LINC01660 show the variability in AT-rich sequences.

**Table 5:** Satellite/Alu/AT-rich regions of LINC01660 (AC011718.2)*.

Start	End	Satellite/Alu /repeat #	Class/family	Repeat
2413	4128	(ATAATAT)n	Simple repeat	
4129	4694	HSATI	Satellite	1
4695	4849	AluYm1	SINE/Alu	
4888	4964	(TA)n	Simple repeat	
4965	5530	HSATI	Satellite	2
5531	5685	AluYm1	SINE/Alu	
5724	6118	(TA)n	Simple repeat	
7047	7159	AluSz	SINE/Alu	

*Data from RepeatMasker analysis of sequence of LINC01660

There are a total of forty repeats of segments of the TBTA in chromosome 22; twenty-four of these repeats are part of the eight lincRNA genes shown in Table 4. Thus, slightly more than half of the total TBTA repeats present in or near the chr 22 22q11.2 region reside in these lIncRNA genes.

### Formation of lincRNA genes

**Fig 5:**
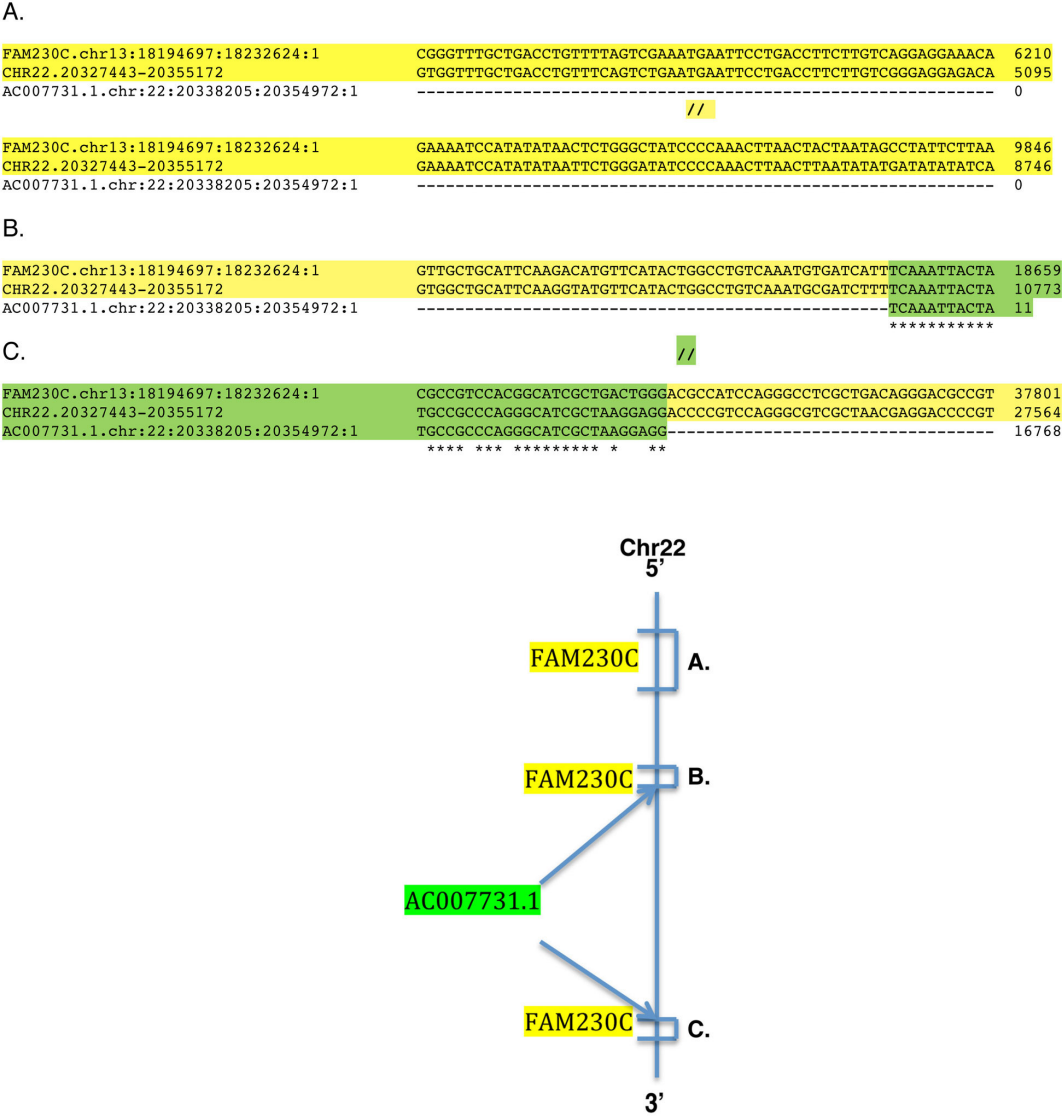
Sequences surrounding gene AC007731.1 on chr 22 Top: Alignment of FAM230C, AC007731.1 and chr 22 sequences. The sequences in sections B. and C. (highlighted in yellow) are outside of but contiguous with the AC007731.1 gene. Bottom: schematic of regions A, B, and C (highlighted in yellow) that represent the close identity of FAM1230C sequences with those of chr22. The sequence in chr 22 that has a high identity with FAM230C and is contiguous with the 5' side of AC007731.1, region B. is ~389 bp long and the FAM230C sequence contiguous on the 3' side of AC007731.1, region C. is ~150 bp. The upstream region of AC007731.1 termed A., consists of 2711 bp segment of chr22 that has a high identity with 5’ half sequences of FAM230C.

Seven of the eight genes of Table 4 (#1–7) are in LCR22 regions associated with chromosomal region 22q11.2. Present also in these regions are segments of the FAM230C sequence that are not part of the lincRNA genes. A sequence similar to the 5’ half of FAM230C is found upstream of the 5’ ends of the lincRNA genes, and there are also sequences with high similarity to FAM230C that are contiguous with lincRNA genes but not part of these genes. For example, there are sequences on chr22 that have a high identity with FAM230C that extend beyond the lincRNA gene AC007731.1 gene at both its 5’ and 3’ terminal ends. Fig 5, top shows an alignment of FAM230C with chr22 and AC007731.1 sequences. Section A. shows the similarity of the 5’ half sequence of FAM230C with the chr22 sequence in a region that is upstream of the gene. Sections B. and C of Fig 5 show contiguous FAM230C sequences with AC007731.1 at its 5’ end (section B.) and its 3’ end (section C.). The bottom schematic of Fig 5 shows a line diagram depicting regions on chr22 that have a high similarity with FAM230C and these are highlighted in yellow. The presence of sequences from the 5’ half of FAM230C that are upstream of lincRNA genes and the sequences that are contiguous with these genes supports the concept that lincRNA genes formed from copies of FAM230C in LCR22 segmental duplications. We hypothesize that these sequences are remnants of duplicated FAM230C that were not incorporated into lincRNA genes during their formation.

In terms of gene composition, there are other sequences that form part of the eight lincRNA genes, in addition to the FAM230C 3’ half sequence. For example, the 3’ end of the AC007731.1 lncRNA gene sequence partially overlaps and is antisense to protein gene USP41 on chr22 and thus shares some USP41 sequences; the remaining and major part of the AC007731.1 sequence consists of FAM230C sequences. In other examples, FAM230B and five other lincRNA genes share a common sequence close to their 3’ ends that extends beyond the region of identity with the 3’ end of FAM230C (Supporting Information, S2 Fig). Thus a combination of different sequences form the eight lincRNA genes, however, they all share the 3’ end sequence of FAM230C and they all have the TBTA/AT-rich motif,

### RNA expression

From the NCBI website that provides gene expression values in human tissues (https://www.ncbi.nlm.nih.gov/guide/genes-expression/) [22], we have been able to outline RNA transcript expression from the lincRNA genes shown in Table 4. Six genes show RNA expression exclusively in the testes (range RPKM 8.9 to 12.9) (Supporting Information, S1 Table; S1 Fig. Data from (https://www.ncbi.nlm.nih.gov/guide/genes-expression/). LINC01658 (AP000345.1) represents a minor exception with low expression in other tissues. Thus there is a stringent specificity in tissue expression from these genes.

### Other ncRNA genes on chr22

A ncRNA gene termed AP000552.3 (ENSG00000237407) has a sequence similar to a short 3’ half segment of FAM230C. This is a small gene of 3185 bp encoded within the sequence of the large lincRNA gene AP000552.1. It is transcribed in the reverse direction from AP000552.1 and on the opposite strand. It has an identity with the antisense sequence strand of FAM230C at nt positions 31657–34832 and its entire sequence consists only of a 3’ segment of FAM230C and does not include the TBTA sequence. Thus this gene is not encoded in a separate locus but within a section of the AP000552.1 gene, and additionally, differs from the eight genes in terms of composition and size. It appears to be in a separate ncRNA gene category.

Another ncRNA gene, FAM230A (NCBI ID 653203 and UCSC:ID uc062bir.1) (chr22:18487127–18500594) contains the very 3’ end section of the FAM230C sequence (nt positions 32771–37928) and does not have the TBTA, however, this gene is believed to produce a nonsense-mediated mRNA decay transcript [33] and thus does not appear to be a lincRNA gene. There is a putative protein gene with the same name, FAM230A (ENSG00000277870, chr 22: 18,422,244-18,500,594) but this gene has a gap of 50,000 bp in an unsequenced region in the middle of the gene; thus, characterization of this putative gene is premature.

### lncRNA genes that have a high similarity to the 5’ end of FAM230C

Importantly, there are lincRNA genes that have an identity only with the 5’ half segment of FAM230C, do not contain the TBTA/AT-rich motif and most reside in chromosomes other than chr22. The most prominent is AP003900.1 ENSG00000277693 on chr21. This lincRNA gene has a high sequence identity to FAM230C (98%) and its entire sequence consists of most of the 5’ half sequence of FAM230C (Supporting Information, S3 Fig).

In other examples, the 5’ half sequence of the FAM230C gene on human chromosome 13 carries a small ncRNA gene within its sequence that produces a reverse strand transcript. This is termed the eukaryotic translation initiation factor 3 subunit F pseudogene2 (EIF3FP2, NCBI ID:838880; Ensemble AL356585.1 ENSG00000279081). The EIF3FP2 gene is 2097 bp long. The gene is situated within the 5’ half of the FAM230C sequence at positions nt 11274–13870 and carries no TBTA sequences. Two closely related genes, EIF3FP1 (NCBI ID: 54053 in chr21 that is encoded within AP003900.1, and EIF3FP3 (NCBI ID: 339799) in chr2 also carry only a segment of the 5’ end sequence of FAM230C, do not have the TBTA sequence and reside in chromosomes outside of chromosome 22.

Additionally, several other pseudogenes also have homology with the 5’ end segment of FAM230C, lack the TBTA/AT-rich motif, and reside in chromosome other than chr22, the homeobox pseudogenes DUXAP 9 (ENSG00000225210) and DUXAP10 (ENSG00000244306) on chr14. Of interest, several disease-related aspects of both DUXAP 9 and 10 have been reported [34–36].

An additional gene that carries a segment of the 5’ end sequence of FAM230C is CECR7. This gene is an exception to the lncRNA genes that have 5’ half sequence of FAM230C in that it is located in chr22. It contains only a small section of the 5’ end of FAM230C, 1937 bp and is situated at chr 22:17036570–17058792, which is far removed from the 22q11.2 region. It is possible that the FAM230C 5’ sequence present in CECR7 originated via transposition of this small sequence and that this gene is not a product of duplication of the FAM230C sequence as the chromosomal region of CECR7 is devoid of other FAM230C sequences. Functions related to CECR7 have recent been shown and they may point to important cancer-related processes [37, 38].

To summarize, with the exception of CECR7, there are six ncRNA genes that carry only 5’ segments of FAM230C, do not have the TBTA motif and are situated in chromosomes other than chromosome 22. In contrast, the eight genes described in Table 4 have copies of the 3’ half of the FAM230C, are found only in chromosome 22, and carry the TBTA.

## Discussion

Palindromic PATRR AT-rich stem loop sequences are found at DNA breakpoints located within the LCR22B segmental duplication in chromosomal region 22q11.2 and several constitutional translocations may involve this region [13, 15]. The eight FAM230C-related genes in LCR22s in chr22 all have AT-rich highly variable sequences, include a large portion of the PATRR sequence and are found in LCR22s, with the presence of one gene in LCR22B. To what extent AT-variable sequences in lincRNA genes may, with further mutations display translocation activity is not known, but some of the TBTA-containing lincRNA gene sequences show long stem loops, and one an almost perfect stem loop structure (see Supporting Information, S4 Fig). Thus one cannot exclude that there may be a potential for breakage with further mutations. It is hypothesized that either long stem loop DNA secondary structures or formation DNA cruciforms are involved in the translocation process [14]. In different but related findings, AT repeats have been found to be one of the most prevalent motifs at DNA translocation breakpoint sites [39].

One concept of why AT-rich and other Pu/Py sequences are stored in lincRNA and/or protein genes is that genes may provide a stability for these motifs, however, this increases the probability of DNA breakage and translocation within these genes, which can alter or inactivate the gene [40–42]. Perhaps this is a consequence of the progression of evolution vis-a-vis the chromosomal translocation process, as mentioned by Bacolla et al [21].

TBTA sequences were previously found in lincRNA gene exons [30]. However, from the current work, these sequences stem from copies of FAM230C sequences carrying the TBTA that are present in these genes. The HSAT1 segment of the TBTA sequence forms the entire sequence of exon1 of several lincRNA genes. This highlights the importance of this repeat unit and of the satellite sequence in lincRNA gene formation and exon composition, and adds another factor, in addition to transposable elements in lncRNA gene formation [43, 44].

Other than chr22, the TBTA motif is not in lncRNA genes present in other chromosomes even though these genes also may have formed from FAM230C duplications in these other chromosomes. For example, chr21 contains repeats of the TBTA/AT-rich motif that are part of a copy of the FAM230C sequence present in chr21, but the TBTA sequences are not incorporated into the lincRNA gene AP003900.1 that was formed from the 5’ end region of the FAM230C copy in chr21. FAM230C sequences without the TBTA segments are also found in lncRNA pseudogenes in chromosomes 2, 9 and 14. There are a relatively small number of genes here, seventeen total FAM230C-related genes, yet they show a pattern. Perhaps cellular regulatory mechanism may secure the formation of TBTA-containing FAM230C-related lincRNA genes only in or near the 22q11.2 region of chr22, but formation of FAM230C-related lncRNA genes without the TBTA in other chromosomes.

In terms of transcription from FAM230C-related lincRNA genes present in or close to the 22q11.2 region, RNA transcripts are found exclusively in human testes with one exception, LINC01658 (AP000345.1) where there is minor expression in other tissues [https://www.ncbi.nlm.nih.gov/guide/genes-expression/; Supporting Information, S1 Table]. The genes outlined in Table 4 may be part of a larger set of lncRNA genes that are exclusively expressed in testes [45].

RNA expression during embryonic development from these and other lncRNA genes in the 22q11.2 deletion region is of interest to assess possible involvement in developmental abnormalities due to a lack of the genes. Ensemble and Expression Atlas have reported RNA expression values for a number of lncRNAs in developing tissues (https://www.ebi.ac.uk/gxa/home/) [46, 47]. Interestingly, the involvement of 22q11.2 lincRNA genes in diseases other than the 22q11.2 deletion syndrome has been shown. For example, the DiGeorge Critical Region 5 (*DGCR5)* lincRNA gene is highly expressed in brain tissue [48] and to a lesser extent in other tissues such as liver. However, RNA expression from this gene is down-regulated in certain diseases: Huntington's disease, where *DGCR5* is regulated by the transcriptional repressor REST [48] and hepatocarcinoma [49].

## Conclusions

The FAM230C gene sequence serves as a source for formation of other lincRNA genes and as a source for spreading of TBTA/AT-rich sequences in chr22. Seventeen lncRNA genes carrying FAM230C sequences have been detected, eight of which contain the TBTA/AT-rich motif. Significantly, the eight genes are all in chr22, localized in or near the critical 22q11.2 deletion region, and all are within low copy repeats, the LCR22 segmental duplications. This work helps define properties of a lincRNA gene family in the chromosomal region 22q11.2 and suggests the mode of lncRNA gene formation of this family.

## Supporting information

S1 Fig LINC01660 RPKM RNA transcript level. Data from NCBI Genes & Expression website: https://www.ncbi.nlm.nih.gov/guide/genes-expression/ Fagerberg et al. [22].
(PDF)

S2 Fig nt sequence alignment of 3' ends of eight lncRNA genes that shows a common sequence shared by six of the eight genes. The positional start site for each gene is close to the end of the identity with FAM230C. Chromosomal coordinates for the common sequence shared by the six genes are 18495612–18500180, Homo sapiens chromosome 22, GRCh38.p7 Primary Assembly.
(PDF)

S3 Fig Alignment of sequences from lincRNA genes FAM230C and AP003900.6 OTTHUMG00000188300 (Ensemble nomenclature AP003900.1 ENSG00000277693). (PDF)

S4 Fig Predicted DNA secondary structure from AT-rich sequence of lncRNA gene AC011718.2 (LINC1660), nt positions 2421–4140. Folding of DNA sequence for secondary structure was with the mFold Web Server: http://unafold.rna.albany.edu/?q=mfold/DNA-Folding-Form Standard conditions (default setting) of folding temperature, ionic conditions and constraint values as were employed. The structure shown below is Structures 1, which represents the lowest delta G value.
(PDF)

S1 Table RPKM (Reads Per Kilobase of transcript per Million mapped reads) for FAM1230C-related genes in chr22. Data compiled from NCBI Genes & Expression website: https://www.ncbi.nlm.nih.gov/guide/genes-expression/ Fagerberg et al. [22].
(PDF)

## Abbreviations

TBTA: Translocation Breakpoint Type A sequence
HSATI: Human satellite I sequences
lncRNA: long non-coding RNA
lincRNAs: long intergenic non-coding RNAs
PATRR: palindromic AT-rich repeat
chr: chromosome
