# Supplementary material for: A family of long intergenic non-coding RNA genes in human chromosomal region 22q11.2 carry a DNA translocation breakpoint/AT-rich sequence
Source: PLoS One. 2018 Apr 18;13(4):e0195702. doi: 10.1371/journal.pone.0195702 (PMC5906017; doi:10.1371/journal.pone.0195702)

A family of long intergenic non-coding RNA genes in human chromosomal region 22q11.2 carry a DNA Translocation Breakpoint/AT-rich sequence

Nicholas Delihas

S1 Fig. LINC01660 RPKM RNA transcript level. Data from NCBI Genes & Expression website: https://www.ncbi.nlm.nih.gov/guide/genes-expression/ Fagerberg et al. [22].

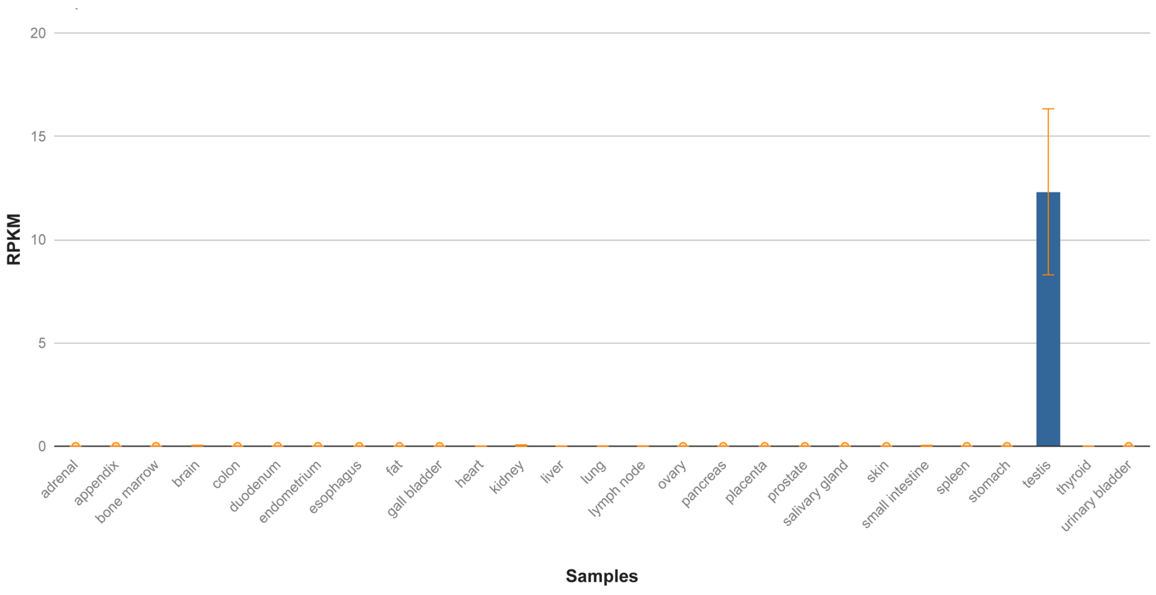

Supplement: S1 Fig — Data from NCBI Genes & Expression website: https://www.ncbi.nlm.nih.gov/guide/genes-expression/ Fagerberg et al. [22]. (PDF) [file pone.0195702.s001.pdf]
