# Supplementary material for: A family of long intergenic non-coding RNA genes in human chromosomal region 22q11.2 carry a DNA translocation breakpoint/AT-rich sequence
Source: PLoS One. 2018 Apr 18;13(4):e0195702. doi: 10.1371/journal.pone.0195702 (PMC5906017; doi:10.1371/journal.pone.0195702)
Supplement: S2 Fig — The positional start site for each gene is close to the end of the identity with FAM230C. Chromosomal coordinates for the common sequence shared by the six genes are 18495612–18500180, Homo sapiens chromosome 22, GRCh38.p7 Primary Assembly. (PDF) [file pone.0195702.s002.pdf]

A family of long intergenic non-coding RNA genes in human chromosomal region 22q11.2 carry a DNA Translocation Breakpoint/AT-rich sequence

Nicholas Delihas

Supporting Information S2 Fig

```
LINC01658.AP000345.1.chr:22:23461486:23487580:-1              --------------------------------ATGTAGACATGAACATCTGCCAATGTGG    16861
LINC01663.chr:GRCh38:22:18872943:18895007:-1                  ------------------------------------------------------------    22065
AC007731.1.chr:22:20338205:20354972:1                         ------------------------------------------------------------    16768
LINC01662.AC008132.15.chromosome:VEGA68:22:18733314:18758506  TGCATTATCGATGTCATCTGCAGTTTAATCAAATGTAGACATGAACATCTGCCAATGTGG    18196
LINC01660.AC011718.2.LINC01660chr:22:18361223:18391705:-1     TGCATTATGGATGTCATCTGCAGTTTAATCAAATGTAGACATGAACATCTGCCAATGTGG    23460
AC008079.1.chromosome:GRCh38:22:18177438:18206515:-1          TGCATTATCGATGTCATCTGCAGTTTAATCAAATGTAGACATGAACATCTGCCAATGTGG    22061
KB-1183D5.13.VEGA68:22:21300390:21325642:-1                   TGCATTATCGATGTCATCTGCAGTTTAATCAAATGTAGACATGAACATCTGCCAATGTGG    18229
FAM230B:VEGA68:22:21167158:21192756:1                         TGCATTATCGATGTCATCTGCAGTTTAATCAAATGTAGACATGAACATCTGCCAATGTGG    18575

LINC01658.AP000345.1.chr:22:23461486:23487580:-1              ACTATTTATGACATCTGCAATTCCCTTGGTGTGGTGCTACTGATTGGCAGCCTCTCATCA    16921
LINC01663.chr:GRCh38:22:18872943:18895007:-1                  ------------------------------------------------------------    22065
AC007731.1.chr:22:20338205:20354972:1                         ------------------------------------------------------------    16768
LINC01662.AC008132.15.chromosome:VEGA68:22:18733314:18758506  ACTATTTATGACATCTGCAATTCCCTTGGTGTGGTGCTATTGATTGGCGGCCTCTCACCA    18256
LINC01660.AC011718.2.LINC01660chr:22:18361223:18391705:-1     ACTATTTATGACATCTGCAATTCCCTTGGTGTGGTGCTATTGATTGGCAGCCTCTCACCA    23520
AC008079.1.chromosome:GRCh38:22:18177438:18206515:-1          ACTATTTATGACATCTGCAATTCCCTTGGTGTGGTGCTATTGATTGGCAGCCTCTCACCA    22121
KB-1183D5.13.VEGA68:22:21300390:21325642:-1                   ACTATTTATGACATCTGCAATTCCCTTGGTGTGGTGCTATTGATTGGCAGCCTCTCACCA    18289
FAM230B:VEGA68:22:21167158:21192756:1                         ACTATTTATGACATCTGCAATTCCCTTGGTGTGGTGCTATTGATTGGCAGCCTCTCACCA    18635

LINC01658.AP000345.1.chr:22:23461486:23487580:-1              ACCCATGCCGGGCACACTGGGGCGTGGTAGATGGCAGCATCCATGATCCACTGCAATGTA    16981
LINC01663.chr:GRCh38:22:18872943:18895007:-1                  ------------------------------------------------------------    22065
AC007731.1.chr:22:20338205:20354972:1                         ------------------------------------------------------------    16768
LINC01662.AC008132.15.chromosome:VEGA68:22:18733314:18758506  ACCCATGCCAGGCACACTGGGGTGTGGTAGATGGCAGCATCCACGATCCACTGCAATGCA    18316
LINC01660.AC011718.2.LINC01660chr:22:18361223:18391705:-1     ACCCATGCCAGGCACACTGGGGTGTGGTAGATGGCAGCATCCACGATCCACTGCAATGCA    23580
AC008079.1.chromosome:GRCh38:22:18177438:18206515:-1          ACCCATGCCAGGCACACTGGGGTGTGGTAGATGGCAGCATCCACGATCCACTGCAATGCA    22181
KB-1183D5.13.VEGA68:22:21300390:21325642:-1                   ACCCATGCCAGGCACACTGGGGTGTGGTAGATGGCAGCATCCACGATCCACTGCAATGCA    18349
FAM230B:VEGA68:22:21167158:21192756:1                         ACCCATGCCAGGCACACTGGGGTGTGGTAGATGGCAGCATCCACGATCCACTGCAATGCA    18695

                                                              //

LINC01658.AP000345.1.chr:22:23461486:23487580:-1              ACTTTTACCTCCTCTCCAAGACAAAACAAACAAAACTAGCATTTTAAAACTTAGTTGTAA    23112
LINC01663.chr:GRCh38:22:18872943:18895007:-1                  ------------------------------------------------------------    22065
AC007731.1.chr:22:20338205:20354972:1                         ------------------------------------------------------------    16768
LINC01662.AC008132.15.chromosome:VEGA68:22:18733314:18758506  ACTTTTACCTCCTCTCCAAGACAAAACAAACAAAACTAGCATTTTAAAACTTAGTTGTAA    24121
LINC01660.AC011718.2.LINC01660chr:22:18361223:18391705:-1     ACTTTTACCTCCTCTCCAAGACAAAACAAACAAAACTAGCATTTTAAAACTTAGTTGTAA    29416
AC008079.1.chromosome:GRCh38:22:18177438:18206515:-1          ACTTTGACCTCCTCTCCAAGACAAAACAAACAAAATTAGCATTTTAAAACTTAGTTGTAA    28018
KB-1183D5.13.VEGA68:22:21300390:21325642:-1                   ACTTTTACCTCCTCTCCCAGACAAAACAAACAAAACTAGCATTTTAAAACTTAGTTGTAA    24186
FAM230B:VEGA68:22:21167158:21192756:1                         ACTTTTACCTCCTCTCCAAGACACAACAAACAAAACTAGCATTTTAAAACTTAGTTGTAA    24532

LINC01658.AP000345.1.chr:22:23461486:23487580:-1              TCTTTCTTCCTTCATGAAAATTTCTCCAACAGCCACTCCCATGGCCCTGTGTGTTCCGGA    23172
LINC01663.chr:GRCh38:22:18872943:18895007:-1                  ------------------------------------------------------------    22065
AC007731.1.chr:22:20338205:20354972:1                         ------------------------------------------------------------    16768
LINC01662.AC008132.15.chromosome:VEGA68:22:18733314:18758506  TCTTTCTTCCTTCATGAAAATTTCTCCAACAGCCACTCCCACGGTCCTGTGTGTTCCGGA    24181
LINC01660.AC011718.2.LINC01660chr:22:18361223:18391705:-1     TCTTTCTTCCTTCATGAAAATTTCTCCAACAGCCACTCCCACGGTCCTGTGTGTTCCGGA    29476
AC008079.1.chromosome:GRCh38:22:18177438:18206515:-1          TCTTTCTTCCTTCATGAAAATTTCTCCAACAGCCACTCCCACGGTCCTGTGTGTTCCGGA    28078
KB-1183D5.13.VEGA68:22:21300390:21325642:-1                   TCTTTCTTCCTTCATGAAAATTTCTCCAACAGCCACTCCCACGGTCCTGTGTGTTCCGGA    24246
FAM230B:VEGA68:22:21167158:21192756:1                         TCTTTCTTCCTTCATGAAAATTTCTCCAACAGCCACTCCCACGGTCCTGTGTGTTCCGGA    24592
```

S2 Fig. Nucleotide sequence alignment of 3' ends of eight lncRNA genes that shows a common sequence shared by six of the eight genes. The positional start site for each gene is close to the end of the identity with FAM230C. Chromosomal coordinates for the common sequence shared by the six genes are 18495612-18500180, Homo sapiens chromosome 22, GRCh38.p7 Primary Assembly.
