# Supplementary material for: A family of long intergenic non-coding RNA genes in human chromosomal region 22q11.2 carry a DNA translocation breakpoint/AT-rich sequence
Source: PLoS One. 2018 Apr 18;13(4):e0195702. doi: 10.1371/journal.pone.0195702 (PMC5906017; doi:10.1371/journal.pone.0195702)
Supplement: S3 Fig — (PDF) [file pone.0195702.s003.pdf]

A family of long intergenic non-coding RNA genes in human chromosomal region 22q11.2 carry a DNA Translocation Breakpoint/AT-rich sequence

Nicholas Delihas

a

```
CLUSTAL O(1.2.4) multiple sequence alignment


FAM230C.chr:13          -------AGAATGGAGTTGAGGTGGGGGCAGCGCTGGACCCCAGGGTCCCTCCCTGCCTC  53
AP003900.6.chr:21       CTGACGCTGAATGGAGTTGAGGTGGGGGCAGCGCTGGACCCCAGGGTCCCTGCCTGCCTC  60
                                ******************************************* ********

FAM230C.chr:13          CTGGGGAGCCCGGTGACCCAGGCAGCCCTGGTGAGGCCGCAGGAGTCTGGGCCCTAGCGA  113
AP003900.6.chr:21       CTGGGGAGCCCGGTGACCCAGGCAGCCCTGGTGAGGCCGCAGGAGTCTGGGCCCTAGCGA  120
                        ************************************************************
```

---

b

```
FAM230C.chr:13          CTTGGGGAGATTGTGGGTTCTCTGATTTCAAGGGGAATGATGTGATCCTAGAGTTGCAAA  7246
AP003900.6.chr:21       CTTGGGGAGATTGTGGGTTCTCTGATTTCAAGGGGAATGATGTGATCCT----------A  7172
                        *************************************************          *

FAM230C.chr:13          GAACAAGTGACAGTGGAGGCGCTTATGCTTTGTGATTGCACTAGAGACAAGGAAGACACA  7306
AP003900.6.chr:21       GAACAAGTGACAGTGGAGGCGCTTATGCTTTGTGATTGCACTAGAGACAAGGAAGACACA  7232
                        ************************************************************

FAM230C.chr:13          ACTAGAATAATGGGGAGCAGGAATGGAGCGGCCAACAGAATATCTGACTGTTAGGGATCT  7366
AP003900.6.chr:21       ACTAGAATAATGGGGAGCAGGAATGGAGCGGCCAACAGAATATCTGACTGCTAGGGATCT  7292
                        ************************************************** *********
```

---

c

```
FAM230C.chr:13          GCCTGACCAACATGGTGAAACCCTGTCTCTACTAAAAATACAAAAATTAGCTGGGTGTGG  15574
AP003900.6.chr:21       GCCAGGCCAACATGGTGAAACCCTGTCTCTACTAAAAATACAAAAATTAGCTGGGTGTGG  15498
                        *** * ******************************************************

FAM230C.chr:13          TGGCACGTGCCTGTAATCCCAGGGACTCAGGAAGCTGAGGCAGGAGAATCGCTTGAACCC  15634
AP003900.6.chr:21       TAGCACGTGCCTGTAATCCCAGTGACTCA-------------------------------  15527
                        * ******************** ******

FAM230C.chr:13          GGCAGGTGGAGGTTGCTGTGAGCTGAGATGGCACCACTCCACTACAGCCTGGTGACAGAG  15694
AP003900.6.chr:21       ------------------------------------------------------------  15527
```

3S Fig. Alignment of sequences from lincRNA genes FM230C and AP003900.6. a. 5' end sequence of AP003900.6, b. Approximate middle part of AP003900.6, c. 3' end of AP003900.6.
