# Supplementary material for: A family of long intergenic non-coding RNA genes in human chromosomal region 22q11.2 carry a DNA translocation breakpoint/AT-rich sequence
Source: PLoS One. 2018 Apr 18;13(4):e0195702. doi: 10.1371/journal.pone.0195702 (PMC5906017; doi:10.1371/journal.pone.0195702)
Supplement: S4 Fig — Folding of DNA sequence for secondary structure was with the mFold Web Server: http://unafold.rna.albany.edu/?q=mfold/DNA-Folding-Form Standard conditions (default setting) of folding temperature, ionic conditions and constraint values as were employed. The structure shown below is Structures 1, which represents the lowest delta G value. (PDF) [file pone.0195702.s004.pdf]

A family of long intergenic non-coding RNA genes in human chromosomal region 22q11.2 carry a DNA Translocation Breakpoint/AT-rich sequence

Nicholas Delihas

S4 Fig. Predicted DNA secondary structure from AT-rich sequence, nt positions 2421-4140 of lncRNA gene AC011718.2 (LINC1660). Folding of DNA sequence for secondary structure was with the mFold Web Server: http://unafold.rna.albany.edu/?q=mfold/DNA-Folding-Form Standard conditions (default setting) of folding temperature, ionic conditions and constraint values as were employed. The structure shown below is Structures 1, which represents the lowest delta G value.

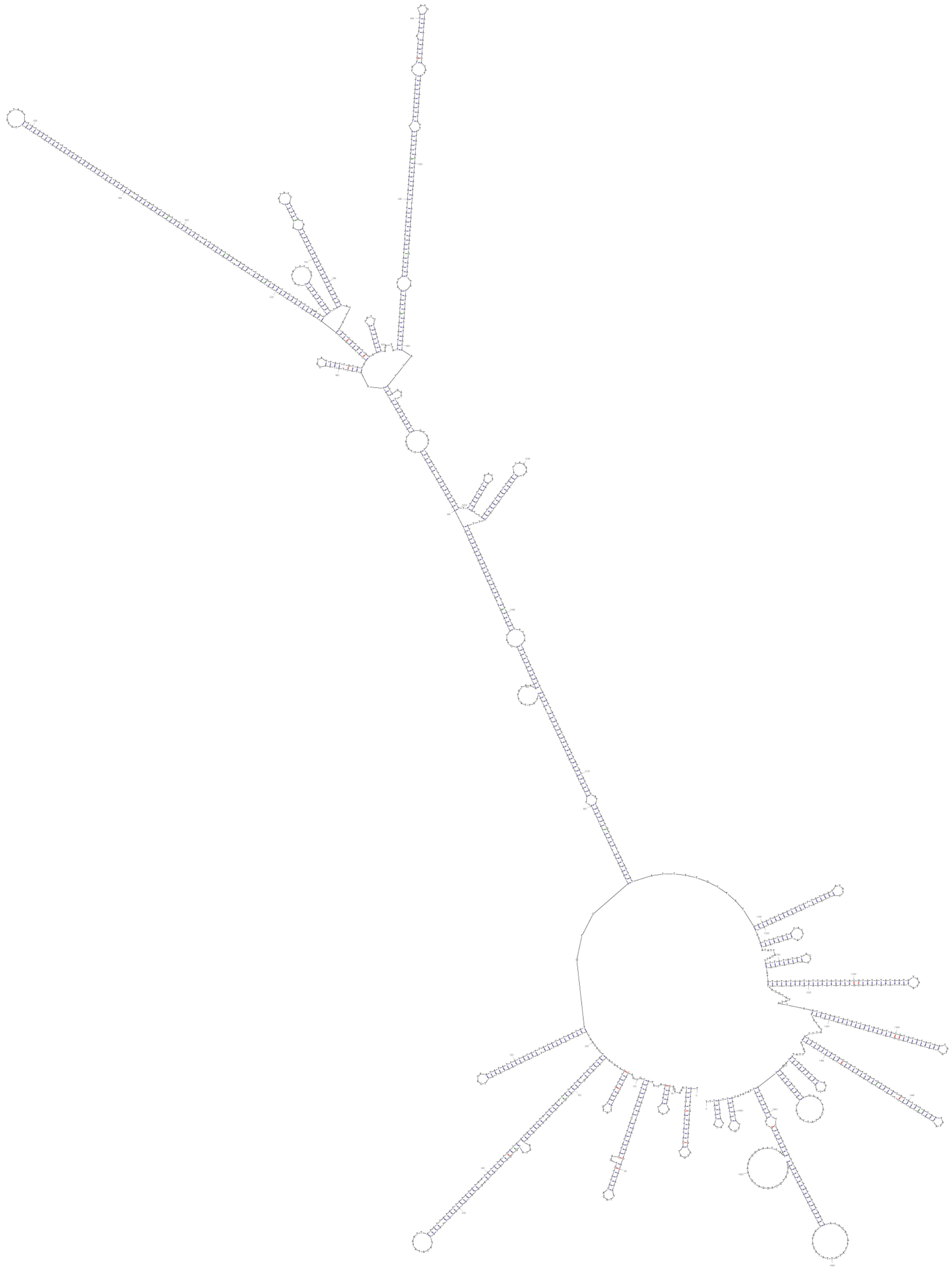

dG = -308.12
