# Supplementary material for: A family of long intergenic non-coding RNA genes in human chromosomal region 22q11.2 carry a DNA translocation breakpoint/AT-rich sequence
Source: PLoS One. 2018 Apr 18;13(4):e0195702. doi: 10.1371/journal.pone.0195702 (PMC5906017; doi:10.1371/journal.pone.0195702)
Supplement: S1 Table — Data compiled from NCBI Genes & Expression website: https://www.ncbi.nlm.nih.gov/guide/genes-expression/ Fagerberg et al. [22]. (PDF) [file pone.0195702.s005.pdf]

A family of long intergenic non-coding RNA genes in human chromosomal region 22q11.2 carry a DNA Translocation Breakpoint/AT-rich sequence

Nicholas Delihas

S1 Table. RPKM (Reads Per Kilobase of transcript per Million mapped reads) for FAM1230C-related genes in chr22

lincRNA gene; chromosomal location; length, LCR22 position	RPKM	Tissue
1. AC008103.3 (LINC01663)	11.9	testes only
chr22: 18,872,943-18,895,007		
22,065 bp; LCR22A		
2. AC011718.2 (LINC1660)	12.3	testes only
chr22: 18,361,823-18,391,105		
30,483 bp; LCR22A		
3. AC008132.15 (LINC01662)	8.9	testes only
Chr22: 18,733,914-18,757,906		
25,913 bp; LCR22A		
4. FAM230B.		
chr 22: 21,167,758-21,192,156	11.1	testes only
25,599 bp; LCR22D		
5. KB-1183D5.13 (AP000552.1)(LOC100996335)	12	testes only
chr22: 21,300,990-21,325,042		
25,253 bp; LCR22D		
6. AC007731.1		
chr22: 20,338,805-20,354,372	undetermined	
16,768 bp; LCR22B		
7. AC008079.1 CC (LOC100996415)	12.9	testes only
chr22: 18,178,038-18,205,915		
29,078 bp; LCR22A		
8. AP000345.1LINC01658.	3.8	testes
chr22: 23,462,086-23,486,980	0.5	prostate
26,095 bp; LCR22#7	0.25	ovary
